# Tragic Optimism as a Buffer Against COVID-19 Suffering and the Psychometric Properties of a Brief Version of the Life Attitudes Scale

**DOI:** 10.3389/fpsyg.2021.646843

**Published:** 2021-09-06

**Authors:** Mega M. Leung, Gökmen Arslan, Paul T. P. Wong

**Affiliations:** ^1^Independent Researcher, Vancouver, BC, Canada; ^2^Department of Psychological Counseling and Guidance, Mehmet Akif Ersoy University, Burdur, Turkey; ^3^International Network on Personal Meaning, Toronto, ON, Canada; ^4^Department of Psychology, Trent University, Peterborough, ON, Canada

**Keywords:** suffering, well-being, COVID-19, existential positive psychology, tragic optimism, Life Attitudes Scale

## Abstract

The Life Attitudes Scale (LAS) was designed to measure tragic optimism (TO)—a distinct type of optimism that could generate hopeless hope even in dire situations according to existential positive psychology (PP 2.0). This study explains why only a faith-based TO could serve as a buffer against suffering at the Nazi death camps as well as the global coronavirus disease 2019 (COVID-19) pandemic. In study 1, the results showed that the factorial structure of a 15-item LAS-Brief (LAS-B), which is a short measure of TO, replicated the original structure of the 32-item long version. The five factors (i.e., affirmation, acceptance, courage, faith, and self-transcendence) provided a good data model fit statistics for LAS-B; the measure had adequate-to-strong internal and latent construct reliability estimates. In study 2, the buffering effect of TO on the association between suffering experiences during COVID-19 and life satisfaction in adults was examined. The results of the studies were consistent with our hypothesis that TO as measured by LAS-B serves as a buffer against the impact of COVID-19 suffering on life satisfaction.

## Introduction

“*Hope springs eternal in the human breast,” and is as necessary to life as the act of breathing—Lewis Latimer*

On March 11, 2020, the WHO declared the outbreak of novel coronavirus 2019 (COVID-19) as a worldwide pandemic (World Health Organization, 2020). Since then, the unpredictable and global spread of the COVID-19 virus had instigated an unsettling multilayered impact on our world, from healthcare crisis to communal lockdowns and eventual economic recession (United Nations Conference on Trade and Development, 2020). As of April 18, 2021, the world has recorded 141,861,840 infection cases and 3,029,583 deaths (Worldometer, 2021). At the early stage of the pandemic, researchers from various parts of the world had already forewarned a pending debacle of the mental health of the public (Cullen et al., 2020; Galea et al., 2020; Rossi et al., 2020). Unfortunately, the early prediction of the researchers became a reality across the world (Canada; Dozois, 2020; United States; Sanderson et al., 2020; India; Banerjee and Bhattacharya, 2020; Kene, 2020; China; Wang et al., 2020; Wu et al., 2020; Italy; Marazziti et al., 2020; Korea; Park and Park, 2020; Chile; Caqueo-Urízar et al., 2020). The surge in psychiatric symptoms was indicative of the pervasive negative impact by the stress brought upon by the pandemic and its ramifications (Brooks et al., 2020; Fitzpatrick et al., 2020; Serafini et al., 2020). Moreover, the prolonged public health measures that include quarantine; physical distancing; and restricted access to entertainment, sports, social meetups, traveling, as well as religious and family gatherings, further tested the limits of his/her coping ability of an individual as a result of the reduced available stress-relief activities, including physical activities (Faulkner et al., 2020) and social support (Marroquín et al., 2020). The negative psychosocial implications of the public health orders have found to increase loneliness and further contributed to the already deteriorating social, mental, and physical wellbeing of the public (Best et al., 2020; Emerson, 2020; Saltzman et al., 2020; Tyrrell and Williams, 2020).

At the time of writing this article, the world was entering its 13th month of the pandemic, and various countries have experienced a second or third wave of the COVID-19 pandemic (Demirtas, 2020; Higgins-Dunn, 2020; Holder et al., 2020; Reuters, 2020). Meanwhile, the public continues to endure the prolonged stress from health threats, financial pressure, and unmet physical, mental, emotional, and social needs as world leaders and policy makers appeal to their citizens to remain optimistic and calm yet vigilant and cautious. The increasing burden on the public has far exceeded the stress level commonly experienced prior to the pandemic. It is therefore essential for individuals to adopt an adaptive mindset that can embrace these seemingly paradoxical demands and an optimistic outlook that can withstand the waves of challenges of this tumultuous time.

### What Kind of Optimism Can Endure During the Pandemic?

The above bleak picture of the scope of distress and suffering during COVID-19 poses a challenge to positive psychology. A research in the positive psychology literature has predominantly examined optimism based on the dispositional optimism model (Scheier and Carver, 1985) and the explanatory style model (Peterson et al., 1982). According to Scheier and Carver, dispositional optimists have a tendency to prescribe favorable outcomes that are independent of their control, effort, or actual facts in relation to the event, also known as generalized outcome expectancies. Alternatively, the explanatory style model posits that optimism/pessimism is based on the tendencies of an individual to attribute the causes of events on three dimensions, namely, internality, stability, and globality. Accordingly, an individual who endorses an optimistic explanatory style would perceive a negative event as (i) externally caused (vs. internal), (ii) unstable in that the situation is temporary (vs. stable), and (iii) relevant in a specific context (vs. global). In the same vein, hope is considered a positive quality that has proven to enhance coping, motivation, and problem-solving. Snyder et al. (1991) defined hope as the positive motivational state based on the successful interaction of an individual's determination and confidence in the pursuit of goals, (i.e., Agency) and the ability to plan routes that lead to the desired outcomes (i.e., Pathways). Hence, in the context of the pandemic, when bad things keep on happening from the global spread of the virus, and from the increasing number of people losing their jobs and having difficulty to provide for their family, is it still possible for people to expect good things to happen most of the time? The global pandemic has lasted more than a year; with the threat of newfound variant strains of the virus spreading exponentially, is it possible for people to dismiss it as a transient incident only limited to a specific place, according to optimistic attribution style? Moreover, when people are dying of COVID-19 in the intensive care unit (ICU), is it still possible for these patients to maintain hope based on self-efficacy?

According to Wong (2009), none of the models of optimism developed by American psychologists is applicable to prolonged traumas like the Holocaust because they are based on a positivity bias toward overestimating his/her agency or unrealistic expectations of an individual that good things will happen. He proposed that only a faith-based tragic optimism (TO) can sustain his/her hope of an individual in situations beyond human control. Frankl (1985) was able to endure unimaginable suffering and the prospect of the gas chamber at the Nazi death camps because he accepted the horrors of living and a tragic sense of life but still affirmed in the intrinsic meaning and values of life and had faith in transforming traumas into human achievement through meaning of self-transcendence. Frankl (1985) concluded that the three basic tenets, namely, freedom of the will, will to meaning, and meaning of life, are capable of transforming the tragic triad of death, suffering, and guilt into TO. This unique kind of optimism has not attracted enough attention from American psychology dominated by an individualistic can-do attitude.

### The History and Development of the Life Attitudes Scale and the Justification of TO as a Unique Kind of Optimism

The initial idea of LAS as a measure of TO was developed in response to 9/11 incident and was first presented in October 2001 at the first International Positive Psychology Summit at the Gallup Center in Washington, DC (Wong, 2001). During that conference, attendees were wrestling with the obvious question: What is the response of positive psychology to 9/11? The consensus was that realistic optimism would be the best answer because the likelihood of such a tragic event would happen again would be less than that of an air traffic accident. The only dissenting voice was Wong (2001) presentation entitled Tragic Optimism, Realistic Pessimism, and Mature Happiness. His presentation advanced the view that 9/11 demanded Frankl's concept of TO in response to Nazi deaths camps. Wong (2001) argued that paradoxically true optimism shined the brightest during the darkest hours; the human spirit grew stronger by embracing a tragic sense of life—with all its inescapable suffering, fragility, and brevity. He further argued that this kind of existential positive psychology would empower people to go on living and enjoy a sustainable mature happiness, no matter how horrible the circumstances are. This less-noticed conference presentation actually laid the foundation for what is later known as existential positive psychology of second wave positive psychology (PP2.0) (Wong, 2010, 2011). Simply put, Frankl (1985) believed that the three basic tenets of logotherapy, namely, freedom of will, will to meaning, and meaning of life, also called the Light Triad, could overcome the tragic triad of death, suffering, and guilt, thus, capable of maintaining optimism in the darkest hours. Wong (2009) conceptualized and operationalized Frankl's concept of TO into five interlocking components, namely, (1) courage to face adversity, (2) self-transcendence (altruism) in serving others, (3) affirmation of the intrinsic meaning of value of life, (4) acceptance of the horrors of being, and (5) faith or trust in God. Wong (2009) proposed that these psychological ingredients constitute the five factors of the optimistic LAS-B, with each factor being measured by five items. Frankl believed that only faith in God or higher power can make the impossible possible, independent of circumstances or our efficacy. Furthermore, faith in the intrinsic meaning and value of life means that as long as we can breathe, life has meaning and everything is possible. With this twofold faith, hope will be as natural as our breathing. Therefore, TO is the most enduring kind of optimism.

The validity of the LAS has been confirmed by a number of MA theses (e.g., Leung et al., 2003). The LAS is a 32-item self-report measure that represents the five components of TO. The four affirmative subscales (i.e., affirmation, courage, faith, and self-transcendence) were designed to measure heroic optimism, with the remaining acceptance subscale designed to assess realistic pessimism. The factorial analyses of the long form LAS supported the dichotomous structure of heroic optimism and realistic pessimism according to the TO model. In addition, the low intercorrelations among the five subscales indicated a relative independence of each subscale representing the five TO components (Leung et al., 2003). Furthermore, the concurrent validity of the LAS was examined with other prominent optimism measures in the literature, in particular, the Life Orientation Test-Revised (LOT-R) (Scheier et al., 1994) and the Adult State Hope Scale (Snyder et al., 1996). The results confirmed the moderate positive correlations between the overall LAS, all four affirmative LAS subscales, the life orientation, and hope. As expected, the acceptance subscale, which denotes the realistic pessimism, was found to be negatively correlated with these variables. It was this unique aspect of TO that made it a unique measure of enduring optimism. A summary of the five studies in the development of the LAS is included in the Appendix.

Recently, Wong (2020a) explained why unrealistic optimism is not adaptive during the pandemic and why the realistic pessimism of acceptance is the key to growing stronger through adversity:

Acceptance is the first step toward personal transformation. The world is full of dangers in the era of COVID-19, regardless of one's rosy worldview. In fact, toxic positivity (Chiu, 2020) and unrealistic optimism (Tong, 2020) may be bad for you during the pandemic according to recent research.

…While such illusions may have some adaptive benefit in ordinary circumstances (Taylor and Brown, 1988; Taylor et al., 2000), it could be very risky in making light of a deadly contagious virus, because no one is immune from the virus, no matter how young and healthy one is. Therefore, it is much better to believe in science rather than positive illusions. Accepting the harsh reality is the first step toward personal transformation (Siegal, 2010).

The results from the study of Mead et al. (2021) further confirmed the promising contributions of TO as a sustainable positive outlook in the context of suffering and adversity. Clinical interventions based on TO have proven to be effective in counseling and trauma treatment in fostering posttraumatic growth (Wong and McDonald, 2002; Leung et al., 2003; Wong, 2010, 2012, 2018; Leung, 2019).

Subjective well-being is conceptualized into two broad components, namely, an affective component (i.e., positive affect and negative affect) and a cognitive/judgmental component (i.e., life satisfaction; Diener et al., 1985). Our study has focused on life satisfaction, which pertains to the conscious cognitive judgment of their perceived life circumstances of individuals compared with a self-imposed set of standards that define a good life (Pavot and Diener, 1993). The examination of life satisfaction was considered more relevant to our study than the affective component as it involves a long-term global evaluation of the perceived success of a person in achieving their values and goals in light of the negative factors in their lives. The general evaluation of the life of an individual which includes both good and bad, i.e., success and failure, aligns with the dualistic view of TO. Hence, it is of interest to determine if TO contributes to the life satisfaction of an individual amid adverse and suffering circumstances such as the current COVID-19 pandemic and its ramifications.

### The Purpose of the Current Study

Although some recent studies confirmed the protective role of dispositional optimism in mitigating the negative impact of the COVID-19 pandemic–related stress on mental health and well-being (Arslan et al., 2020; Arslan and Yildirim, 2021), in the present study, we wanted to test the hypothesis that TO as measured by LAS-B serves as a buffer against the suffering during COVID-19.

First, the aim of study 1 was to investigate the psychometric properties of the LAS-B. While the LAS is a useful measurement in assessing TO, the LAS-B, containing three items for each of the five subscales representing the five components of TO, offers a more convenient administration for clinical and research purposes. Second, based on the extant research and conceptualization of its unique spiritually and existentially oriented attributes, study 2 aimed to explore the protective effect of TO on the association between sufferings during COVID-19 and life satisfaction. It was predicted that TO would significantly contribute to life satisfaction. In addition, it was predicted that TO would buffer against the impact of COVID-19 suffering on life satisfaction.

## Method

### Research Design and Procedures

The current study was designed to collect the experience of individuals in different regions of the world amid the COVID-19 pandemic. Hence, to maximize the inclusivity of various cohort groups across regions within the study timeframe, participants were recruited through the Internet, and the data were collected on an online survey. A URL link of the survey was sent to potential participants through emails and social media within the affiliated networks of the research. In addition, the URL link was included in a post advertised on the Facebook page of International Network on Personal Meaning. The respondents were encouraged to forward the survey to their respective social affiliations and communities that were not affiliated with the researchers to circumvent the response biases from participants who may have personal connections to the researchers. Moreover, participants were informed of the voluntary nature of the study and were assured that no identifiable personal information was collected to ensure anonymity. The data were collected between September 14, 2020, and October 22, 2020.

### Participants

The sample of the study comprised of 215 adults, ranging in age between 18 and 83 years (mean = 47.00, *SD* = 14.07). Participants were 67% females, and approximately 68% of them identified themselves as having a religious affiliation. Moreover, the majority of the participants in the study were from Canada (67%) and the United States (10%).

### Life Attitudes Scale-Brief

The LAS-B is a 15-item self-report measure that represents the five components of TO, which was developed and validated with samples of the present study and the previous validity studies in the development of the LAS (Leung et al., 2003). All items of the LAS-B are scores using 4-point Likert type scale, ranging between 1 (strongly disagree) and 4 (strongly agree). Overall the LAS-B scores are calculated by summing all the responses. The reliability and validity of the measure are presented in the “Results” section.

### Suffering Measure During COVID-19

Suffering Measure during COVID-19 (SM-COVID-19) was used to measure the coronavirus-related suffering experiences of people (Wong, 2020b). The measure is a 10-item self-report scale (e.g., “poor physical health condition”), and all items were scored using a 5-point Likert-type scale, ranging from 1 (not at all) to 5 (a great deal). Arslan et al. (2021) reported that the scale had adequate data model fit statistics and strong internal reliability estimates with adults. The internal reliability estimate of the scale with the present study was strong (see **Table 2**).

### Satisfaction With Life Scale

The Satisfaction with Life Scale (SWLS) was used to measure the life satisfaction of people (Diener et al., 1985). The SWLS is a 5-item self-report scale (e.g., “The conditions of my life are excellent”), and all items are responded using a 7-point Likert-type scale, ranging from 1 (strongly disagree) to 7 (strongly agree). Previous studies indicated that the scale had strong internal reliability estimates (Diener et al., 1985). The internal reliability estimate of the scale with the present study was strong (see **Table 2**).

### Data Analyses

A two-step analytic approach was used to generate the items for LAS-B. In the first step, the construct validity of the scale, which was described in the previous development and validity study of LAS (Leung et al., 2003), was examined using the confirmatory factor analysis with the sample of the development study (*N* = 366; see Leung et al., 2003, for more information). Three items for each subscale were selected based on the following criteria: the largest factor loadings, uncorrelated error variables, smallest cross-loadings, and item-total correlations (Leite et al., 2008). The items with the highest factor loading on a given subscale were specified for that subscale, and other rules (e.g., uncorrelated error variables and smallest cross-loadings) were then checked. Using these criteria, three items of each subscale were selected to form the LAS-B, which, in turn, was subjected to another confirmatory factor analysis with the second sample of the study. Several common model fit statistics and their cutoff values were examined to assess the following results of the construct validity: Tucker–Lewis index (TLI ≥ 0.95 for good fit and ≥0.90 for acceptable fit), comparative fit index (CFI ≥ 0.95 for good fit and ≥0.90 for acceptable fit), the root mean square error of approximation (RMSEA ≤ 0.06 for good fit and ≤ 0.10 for acceptable fit), and the standard root mean square residual (SRMR ≤ 0.06 for good fit and ≤ 0.10 for acceptable fit; Hooper et al., 2008; Kline, 2015). The reliability analyses were also conducted using the internal (α) and latent construct (*H*) reliability estimates, and the findings from these analyses were evaluated using their cutoff scores: reliability estimate ≥0.60 = adequate and ≥0.70 = acceptable (Carter et al., 2009; Gaskin, 2016). In the second step, the moderating effect of TO on the association between COVID-19-related suffering and life satisfaction was examined using the PROCESS macro (Model 1) for the SPSS application (Hayes, 2018). All analyses were conducted using PROCESS macro version 3.5, AMOS version 24, and SPSS version 25 software.

## Results

The findings from the first confirmatory factor analysis showed that the measurement model, which structured each of the 32 items as indicators of the five latent constructs (i.e., affirmation of meaning and value, acceptance, courage, faith, and self-transcendence), yielded poor-to-adequate data model fit statistics—χ^2^ = 1,313.90, *df* = 454, *p* < 0.001, CFI = 0.81, TLI = 0.79, RMSEA (95% confidence interval [CI]) = 0.072 (0.068, 0.077). Factor loadings of the measurement model were generally adequate to strong (affirmation of meaning and value λ range = 0.45–0.79; acceptance λ range = 0.35–0.60; courage λ range = 0.38–0.56; faith λ range = 0.48–0.88; and self-transcendence λ range = 0.41–0.69).

**Figure 1:**
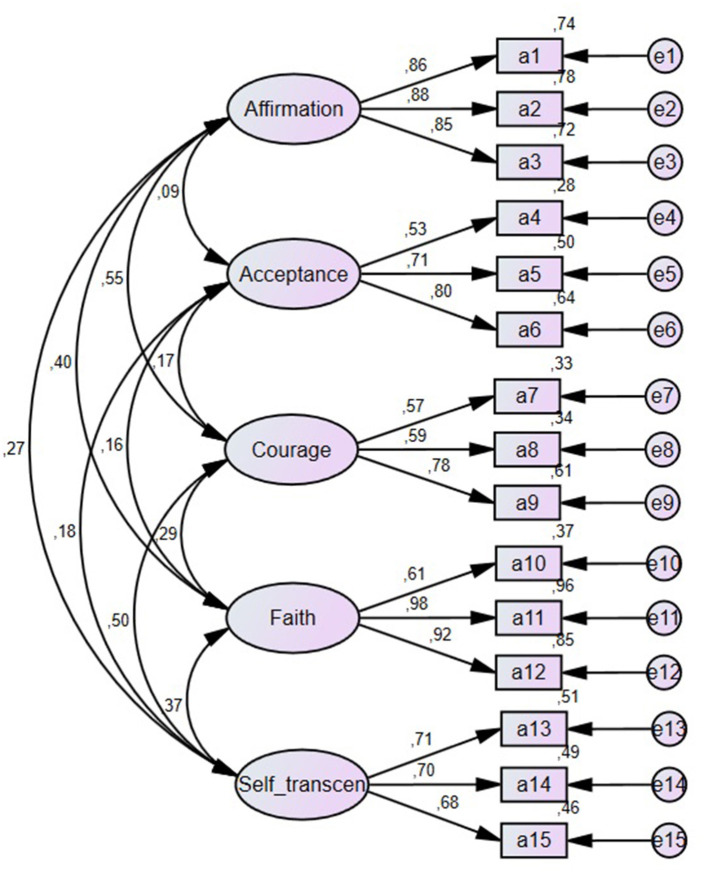
Confirmatory factor analysis results indicating standardized regression estimates.

Based on this factor analysis, three items with the highest factor loading were selected for each subscale (as shown in Table 1), and the confirmatory factor analysis, which was conducted to examine the construct validity of the measure, was rerun to test the LAS-B model, which structured each of the 15 items as indicators of the five latent structures, with the sample of the study. The findings from the analysis indicated good data model fit statistics—χ^2^ = 153.47, *df* = 80, *p* < 0.001, CFI = 0.95, TLI = 0.93, RMSEA (95% CI) = 0.066 (0.050, 0.081), and factor loadings of the model were strong, ranging between 0.53 and 0.98, with robust indicator reliabilities (ℓ^2^), as shown in Table 1 and Figure 1.

**Table 1:** Scale items, internalizing domains, and factor loadings.

**Scales and items**	**CFA factor loadings**				
	****λ_1_****	**$\ell_{1}^{2}$**	****λ_2_****	**$\ell_{2}^{2}$**	***H***
Affirmation of meaning scale	—	—	—	—	0.90
My life is worth living no matter how many problems I have	0.79	0.63	0.86	0.74	—
Even if I were stripped of everything, I still believe that my life is precious	0.74	0.55	0.88	0.78	—
Life is worth living no matter how difficult or painful it is	0.69	0.48	0.85	0.72	—
Acceptance scale	—	—	—	—	
My life is fragile, and could end at any time	0.42	0.17	0.53	0.28	0.76
Life is full of setbacks	0.55	0.31	0.71	0.50	—
It is inevitable that people will let me down	0.60	0.36	0.80	0.64	—
Courage scale	—	—	—	—	0.72
I can move forward with confidence, even if most people don't approve of my life goals	0.49	0.17	0.57	0.33	—
I'd rather die fighting for something I believe in than play safe	0.46	0.33	0.59	0.34	—
I am willing to face horrible consequences in order to do what is right	0.56	57	0.78	0.61	—
Faith scale	—	—	—	—	0.96
I believe in a higher power	0.84	0.70	0.61	0.37	—
Putting my fate in God's hands has helped me gain peace in my life	0.86	0.74	0.98	0.96	—
Even when I am at the end of my rope, I still believe that God will come to my rescue	0.88	0.77	0.92	0.85	—
Self-transcendence scale	—	—	—	—	0.74
Living for others helps me rise above my own problems	0.69	0.48	0.71	0.51	—
My suffering decreases whenever I reach out to help others	0.68	0.46	0.70	0.49	—
Being an example to others motivates me to endure hardships	0.62	0.38	0.68	0.46	—

*CFA, confirmatory factor analysis; λ_1_, item loadings for first-order factors; $\ell_{1}^{2}$, indicator reliability for first-order factor items (N = 366); λ_2_, factor loading for the first-order structure; $\ell_{2}^{2}$, indicator reliability for first-order factor indicators (N = 215); H = latent construct reliability*.

The latent structure of the scale descriptive statistics was examined for the LAS-B, the SM-COVID-19, and the SWLS. The results showed that the skewness and kurtosis scores of these three measures ranged between −1.40 and 2.39, suggesting that all three measures had relatively normal distributions, as shown in Table 2. The findings from the study also revealed that all scales of the LAS-B had acceptable-to-strong internal and latent construct reliability estimates (α range = 0.67–0.89; *H* range = 0.72–0.96).

**Table 2:** Descriptive statistics of the measure of the study.

**Scales**	***Mean***	***SD***	**Skewness**	**Kurtosis**	**α**
LAS-B total scores	46.63	6.38	−0.61	1.45	0.81
Affirmation of meaning	10.33	1.88	−1.40	2.39	0.89
Acceptance	8.79	2.15	−0.20	−0.73	0.70
Courage	8.73	1.73	−0.16	0.09	0.67
Faith	9.50	2.65	−0.91	−0.23	0.87
Self-transcendence	9.26	1.65	0.14	−0.60	0.74
Suffering during COVID-19	23.99	8.05	0.51	−0.23	0.89
Subjective well-being	23.60	6.10	−0.73	0.38	0.78

*COVID-19, coronavirus disease 2019; LAS-B, Life Attitudes Scale-Brief*.

**Figure 2:**
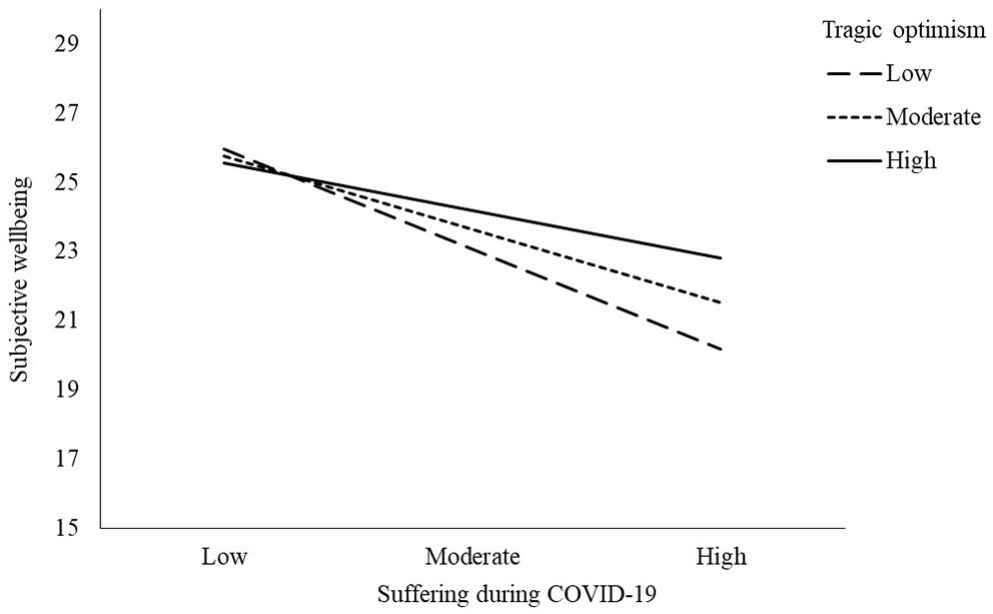
Moderating effect of tragic optimism.

We secondly aimed to examine whether TO moderated the association between suffering during COVID-19 and life satisfaction. Before testing the model, the correlation analysis was conducted to examine the association between the study variables. The results from the correlation analysis indicated that suffering had a significant and negative association with life satisfaction (*r* = −0.41, *p* < 0.001), but the association between suffering and TO was not significant (*r* = −0.10, *p* = 0.129). We also found that there was no significant relationship between TO and life satisfaction (*r* = 0.13, *p* = 0.051). After controlling for the demographic variables (e.g., age, gender, and having religious affiliation), the findings from the moderation analysis revealed that suffering significantly and negatively predicted life satisfaction, whereas TO was a non-significant predictor of life satisfaction. The interaction effect between suffering and TO on life satisfaction was significant, accounting for 2% of the variance in life satisfaction. The moderation model explained 22% of the variance in life satisfaction, as shown in Table 3. Additionally, the simple slope effect indicated that the effect of suffering on life satisfaction was observed when TO was high (+1 *SD*), moderate, and low (−1 *SD*), as shown in Figure 2. These results indicated that TO serves as a protective factor on the life satisfaction and mental health of people in the context of adverse life experiences.

**Table 3:** Results from a moderation analysis.

		**Coeff**.	***SE***	***t***	***p***
Constant		20.43	2.18	9.7	<0.001
Suffering (*X*)		−0.26	0.05	−5.839	<0.001
Tragic Optimism (*W*)		0.09	0.07	1.26	0.210
Suffering x Tragic Optimism (*XW*)		0.02	0.01	2.019	0.03
Age		0.01	0.03	0.11	0.908
Gender		1.80	0.81	2.21	0.028
Religious affiliation		0.38	0.98	0.39	0.695

*R^2^ = 0.122, MSE = 29.93.*

*F_(6, 2, 105)_ = 9.49, p < 0.001*.

## Discussion

The aim of study 1 was to develop and validate the LAS-B, which structured each of the 15 items as indicators of the five latent structures (i.e., affirmation of meaning and value, acceptance, courage, faith, and self-transcendence), among the samples of adults. The findings from the confirmatory factor analysis indicated a good data model fit statistics, and factor loadings of the measure were strong, ranging between 0.53 and 0.98, with robust indicator reliabilities. Further results also revealed that all subscales of the LAS-B had acceptable-to-strong internal and latent construct reliability estimates. Consistent with the long version of the LAS (Leung et al., 2003), these results suggest that the brief version of the measure had psychometrically adequate properties for use in assessing TO among adults.

The aim of study 2 was to explore the buffering effect of TO on the adverse impact of suffering on the life satisfaction of people in the context of the COVID-19 pandemic. Results from the moderation analysis indicated that suffering during COVID-19 had a significant and negative predictive effect on life satisfaction, whereas TO was a non-significant predictor of life satisfaction. TO moderated the impacts of suffering during COVID-19 on life satisfaction and served as a buffer against its adverse impacts. The significant negative predictive effect of suffering during COVID-19 on life satisfaction was expected due to the challenges and hardships posed by the pandemic ramifications (i.e., job loss and the restrictions to social activities), which lowers the well-being as a result of pleasure deficits and elevated distress emotions (Kahneman et al., 1999). However, contrary to the hypothesis of the research team that TO would significantly contribute to life satisfaction, the results indicated that there was no significant relationship between TO and life satisfaction and that TO was a non-significant predictor of life satisfaction. This statistically insignificant relationship between TO and life satisfaction can be explained by the use of SWLS, which measures hedonic happiness (Kahneman et al., 1999) rather than mature happiness (Wong and Bowers, 2018), which is the more sustainable happiness during suffering. Aligned with the prediction that TO would buffer against the impact of COVID-19 suffering on life satisfaction, the interaction effect between suffering and TO on life satisfaction was found to be significant. These results provide evidence indicating that TO serves as a protective factor on the life satisfaction of people in the context of suffering during the COVID-19 public health crisis.

More importantly, the present research on durable optimism based on accepting suffering further contributes to the growing literature of the new science of PP2.0. Any equation of happiness and well-being must factor in the misery index of macro stressors, such as natural disasters and pandemics, and micro factors, such as personal limitations, painful memories, or emotions (Fowers et al., 2017; Wong, 2019, 2020a; Van Tongeren and Van Tongeren, 2020). Indeed, it is not possible for human beings to remain resilient and optimistic without factoring in suffering, the inescapable reality of human existence (Joye, 2011; Bates, 2016; Wong, 2019). The present study also indirectly supports the importance of self-transcendence in well-being and resilience, which already has considerable empirical support (Wong, 2016; Kaufman, 2020; Wong et al., in press). By reframing the distressing aspects of the COVID-19 in an altruistic and prosocial perspective, a spiritual transformation and shift in perception from his/her own suffering of an individual to a wider vision that embodies empathy for the suffering of others can occur (Geppert and Pies, 2020). We can conclude that in future research, especially in the age of COVID-19, positive psychology research on well-being, optimism, or flourishing needs to take into account the suffering as a covariable or statistical covariate, a main tenant of the new science of existential positive psychology (PP2.0).

### Implications and Limitations

One implication is regarding the relationship between optimism and hope. Historically, these two constructs have been used interchangeably. Recently, Fowler et al. (2017) confirmed that though the two shared common properties that pertain to global expectancy, they were indeed distinct constructs as indicated by their unique associations with other measures. Thus, their findings supported that optimism and hope are best conceptualized by a bi-factor model, which consists of both a global and a distinct two-factor component such that the two processes serve as different strategies to ensure success in goal attainment. Snyder et al. (1991) proposed that “the high-hope person's analysis of sufficient agency and pathways in a given goal setting should lead to the perception of the relatively high probability of goal attainment” (p. 571). Thus, his hope theory is very similar to the theory of efficacy suggested by Bandura (Peterson, 2000) based on the belief in his/her agency of an individual. Lazarus (1999) argued that hope was a coping resource against despair, and he considered hope as “yearning for amelioration of a dreaded outcome” (Lazarus, 1991, p. 282) in a stressful situation. Bury et al. (2016) considered optimism as being directly related to the positive expectation of an outcome, while hope is related only to the possibility of an outcome with personal of collective investment of effort. From the Judeo-Christian perspective, hope is strongly related to faith, “Now faith is confidence in what we hope for and assurance about what we do not see” (Hebrew 11:1 NIV).

TO is primarily an attitude or life orientation in a world full of dangers and suffering as during COVID-19. It is hopeful that eventually things would turn out OK, but this positive expectation is based on faith in God and not on his/her own agency and efforts of an individual. Thus, only TO can provide a glimpse of hope in hopeless situations, such as buried three miles underground in a mine or dying of COVID-19 or cancer in an ICU. According to the resource-congruent model of coping suggested by Wong et al. (2006), TO, as a form of religious and existential coping, is the most adaptive response in situations beyond human control.

### Clinical Implications of TO and LAS-B

The five interlocking components of the TO model, namely, affirmation, courage, faith, self-transcendence, and acceptance correspond to the ABCDE strategy of the Meaning Therapy of Wong, which targets to help individuals achieve a dialectical balance of embracing the negative aspects and deepening appreciation for the positive aspects of life (Wong, 2010, 2012). The clinical implications of the ABCDE strategy and TO in trauma therapy were previously examined (Leung, 2019). For clinical purposes of the LAS-B, the administration of this measure offers a convenient way to assess the state of the existential well-being of an individual. The responses on the LAS-B items allow practitioners to determine the strengths and coping resources of an individual in relation to his/her faith and existential beliefs. Likewise, low scores on the subscale(s) may indicate areas of focus in treatment. The TO model has proven to be effective in trauma therapy, and the same applicability pertains to individuals suffering in the COVID-19 pandemic. It is not an understatement to say that the pandemic is a global trauma. As such, the dichotomous structure of TO allowing the co-existence of conflicting emotions and experiences offers valuable insight for coping with the COVID-19 suffering. Aligned with the PP 2.0 framework, TO affirms that over the course of the pandemic and even post pandemic, the presence of both positive and negative emotions is valid and normal. A person could grieve for the loss of certain aspects of life due to the pandemic and, at the same time, celebrate the joyful moments concurrent with the grief. Thus, it is particularly important for individuals to adopt a balanced perception to validate all aspects of their experiences as only an integrated outlook on life fosters maturity and posttraumatic growth (Leung et al., 2003; Leung, 2019). The applications of the TO components in relation to coping with the COVID-19-related suffering are briefly discussed as follows:

1. Accept that losses, frustration, and grief are inevitable during this time and acknowledge the inherent vulnerability, uncertainty, and instability of the circumstance (acceptance of what cannot be changed).
2. In spite of the frustrating, disheartening, and worrisome situation, affirm that life is worth living and focus on the appreciation for the meaningful aspects of life such as relationships with loved ones, health, possibilities, goodness, and wonders of life (affirmation of the meaning and value of life).
3. Having faith in God or a higher power, regardless of religious affiliation, enhances the ability to endure suffering as one finds solace trusting that a greater being, transcendental to the chaotic state of the world, remains in control and that order and healing will be restored (faith and trust in God or a higher power).
4. Take courage and remain steadfast in his/her personal responsibilities, goals, and purpose despite challenging circumstances (courage to face adversity).
5. During disasters or turbulent times, the most effective adaptive response is to help others selflessly and serve the common good. We can get through the pandemic better, if we are all willing to sacrifice some personal liberty (such as staying home and wearing masks) for the greater good of preventing the spread of the virus (self-transcendence simply means to serve something greater than oneself).

There are limitations to the study that may affect the generalization of the results. First, due to the time sensitivity of the study, a cross-sectional sampling method limited to one wave was used rather than a prospective study with several waves. Second, the convenience sample for the study comprised of the participants primarily residing in North America (i.e., 67% in Canada and 10% in the United States), which may not be representative of the populations in other parts of the world with varying experiences of the COVID-19 pandemic. Third, the study was conducted online and that adequate skill of usage of a computer or mobile device was necessary to participate; hence, the results may be biased toward younger cohorts who are more adept at using computers or mobile devices and with a more convenient access. Fourth, it is acknowledged that subjective well-being is multidimensional and is comprised of an affective and a cognitive/judgmental component. Our study focused only on the cognitive component (i.e., life satisfaction). Future studies could examine both the cognitive and affective components to comprehensively understand the effects between TO, subjective well-being, and COVID-19-related suffering. Lastly, the results were collected based on self-report measures, which may contain inherent biases. Future studies should also use objective and behavioral measures to increase the accuracy of the results.

## Conclusion

The dualistic infrastructure of the TO model serves as a prototype of existential positive psychology that embraces both positive affirmation and expectation, as well as the dark side of life from the lens of existentialism (Wong, 2021). TO offers a distinctive type of optimism that can sustain hope in spite of dire circumstances such as the current COVID-19 pandemic era. Moreover, the LAS-B that measures the five TO components, namely, affirmation, courage, faith, self-transcendence, and acceptance, can be employed for clinical and research purposes to assess the existential based resilience amid adverse conditions. The results of the LAS-B can be explored to enrich future research on optimism and well-being in the context of suffering.

## Data Availability Statement

The raw data supporting the conclusions of this article will be made available by the authors, without undue reservation.

## Ethics Statement

The studies involving human participants in study 1 were reviewed and approved by Trinity Western University. All procedures performed in the studies involving human participants were in accordance with the ethical standards of the institutional and/or national research committee and with the 1964 Helsinki declaration and its later amendments or comparable ethical standards. The participants provided their written informed consent to participate in this study. Ethical review and approval was not required for study 2 on human participants in accordance with the local legislation and institutional requirements.

## Author Contributions

ML was the main research coordinator of the study and contributed to writing the introduction and discussion of the manuscript. GA contributed to all steps of the research process, ran the analysis, and contributed to writing the manuscript. PW oversaw the study and contributed to writing the introduction and discussion of the manuscript. All authors contributed to the article and approved the submitted version.

## Conflict of Interest

The authors declare that the research was conducted in the absence of any commercial or financial relationships that could be construed as a potential conflict of interest.

## Publisher's Note

All claims expressed in this article are solely those of the authors and do not necessarily represent those of their affiliated organizations, or those of the publisher, the editors and the reviewers. Any product that may be evaluated in this article, or claim that may be made by its manufacturer, is not guaranteed or endorsed by the publisher.
