# Supplementary material for: Tragic Optimism as a Buffer Against COVID-19 Suffering and the Psychometric Properties of a Brief Version of the Life Attitudes Scale
Source: Front Psychol. 2021 Sep 6;12:646843. doi: 10.3389/fpsyg.2021.646843 (PMC8450366; doi:10.3389/fpsyg.2021.646843)
Supplement: Supplementary file 1 [file Data_Sheet_1.pdf]

Appendix

The development and validation of the Life Attitudes Scale (Leung et al., 2003) consisted of five separate studies.

Study 1 – Item Generation

The research team generated 97 items based on the five TO constructs, which were reduced to 42 items refined for clarity of expression. The 42 items were given to 8 judges to sort according to the five TO constructs based on Face validity. The 42 items were administered online to 231 Participants recruited through Email, Word of Mouth, and the International Network on Personal Meaning (INPM) website. A Cronbach's alpha was performed on all 42 items followed by a Principal Components Analysis (PCA) utilizing an orthogonal (Varimax) rotation. The analysis produced 10 factors with eigenvalues greater than one. Seven of the ten factors were interpretable and the other three factors were eliminated because they were not interpretable. Items with a loading lower than .4 on all components or double loaded on more than one component were eliminated. Another PCA was conducted to a seven-factor solution. Consequently a total of 15 items were eliminated; thus, the original list of 42 items was refined to 27 items. With the 27 items retained from the initial pool, some of the seven factors were defined by less than three items. Therefore, to increase the internal consistency, 9 additional items were generated to ensure that each subscale had a minimum of four items. The additional items resulted in a 36-item scale.

Study 2 – Item Selection & Analysis

The 36 items were administered to 183 participants online using the same recruitment methods as the previous study. A PCA was repeated on the 36 items with orthogonal rotation.

The analysis produced a seven-factor solution instead of the predicted five-factor because two of the five theoretical subscales, Courage and Acceptance, each split into two factors thus creating a total of seven factors. Among the 36 items, 35 loaded greater than .4 on a single factor and one item loaded greater than .4 on more than one factor. A total of four items were eliminated (the double-loading item and three items that did not load on their conceptually defined components). The resulting scale of 32 items is hereafter referred as the Life Attitudes Scale (LAS).

Scale-Level Principal Components Analysis on Subscale

A PCA was conducted on the subscale level to demonstrate the theoretical structure of the LAS as consisting of two domains: Idealistic optimism and Realistic pessimism. The results showed that the four optimism subscales of the LAS: Affirmation of Meaning and Life, Courage, Faith and Self-Transcendence loaded highly on component one which represented Idealistic optimism, while the Acceptance subscale loaded highly on component two, which represented Realistic pessimism; thus the results confirmed the dichotomous structure of the TO model.

Internal Consistency

The internal consistency of the LAS was measured using Cronbach's alpha on both the subscale level as well as the global scale level. All subscales and the global scale demonstrated moderate to high internal consistency with alpha coefficients ranging from .61 to .94.

Studies 3 & 4 – Concurrent Validity of LAS

In Study 3, the 32-item LAS, Life Orientation Test –Revised (LOT-R) and Adult State Hope Scale were administered to 214 participants online using the same recruitment methods as the previous studies whereas the LAS and the Personal Meaning Profile (PMP) were administered to 30 participants in Study 4. The concurrent validity of the LAS was examined in

comparison to other optimism measures: LOT-R (i.e. dispositional optimism) and the Adult State Hope Scale (i.e. self-efficacy based optimism); likewise the LAS was compared to the PMP to determine its basis on meaning and faith. Correlation analyses and partial correlation analysis controlling for the Acceptance subscale were conducted. Results indicated that the LAS demonstrated convergent validity with other optimism measures as well as with the Personal Meaning Profile. Factorial analyses were repeated in Study 3. A PCA utilizing Varimax rotation based on the 214 participants sample was repeated on the 32 items and a five-factor solution was imposed. All 32 items of the LAS fell into a priori theoretical categories with only minor deviations. Likewise, a PCA was repeated on the subscale level and results confirmed the dichotomous structure of the LAS with its four optimism subscales loaded on one component and the acceptance subscale loaded on the second component. The subscale structure was considered stable.

Study 5 – Predictive Validity of LAS

The LAS, the Posttraumatic Stress Growth Inventory (PTGI) and the Stressful Life Experiences Screen (SLES) were administered to 153 participants. To establish the predictive validity of the LAS on Posttraumatic growth, Pearson product-moment correlations were calculated between the scores of the LAS, the PTGI, as well as the SLES. In addition to the existing three subscales on the Stressful Life Experiences Screen, an index (SLES diff) calculated by the difference between the scores on the Stressfulness Then and Stressfulness Now subscales was included as a quantitative measure of posttraumatic change. A correlation analysis on the global LAS score, PTGI and SLES diff was conducted. Results indicated that LAS is a predictor of Posttraumatic growth. A Hierarchical Regression analysis was then conducted to

determine whether TO functions as a mediator or moderator in the Trauma-Post traumatic growth relationship.

Finally, the analyses of the LAS factorial structure, subscale structure, and internal consistency were replicated. A PCA was repeated on the LAS factorial structure utilizing a different sample (n=153). A five-factor solution was imposed on the factorial structure to determine the factorial stability of the scale, followed by a PCA on the subscale level. Results were similar to the findings from Studies 2 & 3 that confirmed most LAS items loaded according to the five factors and that four of the LAS optimism subscales loaded highly on one component with the acceptance subscale loaded highly on a separate component. From the results in Studies 2, 3, and 5, the overall factorial structure and the subscale structure of LAS were proven to be highly stable and the internal consistency of the scale remained moderate to high across the different samples.
